# A horizon scan of global conservation issues for 2014

**DOI:** 10.1016/j.tree.2013.11.004

**Published:** 2014-01

**Authors:** William J. Sutherland, Rosalind Aveling, Thomas M. Brooks, Mick Clout, Lynn V. Dicks, Liz Fellman, Erica Fleishman, David W. Gibbons, Brandon Keim, Fiona Lickorish, Kathryn A. Monk, Diana Mortimer, Lloyd S. Peck, Jules Pretty, Johan Rockström, Jon Paul Rodríguez, Rebecca K. Smith, Mark D. Spalding, Femke H. Tonneijck, Andrew R. Watkinson

**Affiliations:** 1 Conservation Science Group, Department of Zoology, Cambridge University, Downing Street, Cambridge, CB2 3EJ, UK; 2 Fauna & Flora International, 4th Floor, Jupiter House, Station Road, Cambridge, CB1 2JD, UK; 3 International Union for Conservation of Nature, 28 rue Mauverney, CH-1196 Gland, Switzerland; 4 Centre for Biodiversity and Biosecurity, School of Biological Sciences, University of Auckland, PB 92019, Auckland, New Zealand; 5 Natural Environment Research Council, Polaris House, North Star Avenue, Swindon, SN2 1EU, UK; 6 John Muir Institute of the Environment, The Barn, One Shields Avenue, University of California, Davis, CA 95616, USA; 7 Royal Society for the Protection of Birds, The Lodge, Sandy, SG19 2DL, UK; 8 WIRED, 520 3rd Street, Third Floor at Bryant Street, San Francisco, CA 94107, USA; 9 Centre for Environmental Risks and Futures, Cranfield University, Cranfield, MK43 0AL, UK; 10 Natural Resources Wales, Cambria House, 29 Newport Road, Cardiff, CF24 0TP, UK; 11 Joint Nature Conservation Committee, Monkstone House, City Road, Peterborough, PE1 1JY, UK; 12 British Antarctic Survey, Natural Environment Research Council, High Cross, Madingley Road, Cambridge, CB3 0ET, UK; 13 Essex Sustainability Institute and Department of Biological Sciences, University of Essex, Colchester, CO4 3SQ, UK; 14 Stockholm Resilience Center, Stockholm University, Kräftriket 2B, SE-106 19, Stockholm, Sweden; 15 Center for Ecology, Venezuelan Institute for Scientific Investigation (Instituto Venezolano de Investigaciones Científicas — IVIC), Apdo. 20632, Caracas 1020-A, Venezuela; 16 Global Marine Team, The Nature Conservancy, Department of Zoology, Cambridge University, Downing Street, Cambridge, CB2 3EJ, UK; 17 Wetlands International, PO Box 471, 6700 AL Wageningen, The Netherlands; 18 School of Environmental Sciences, University of East Anglia, Norwich, NR4 7TJ, UK

**Keywords:** climate change, diseases, future, horizon scan, priority setting

## Abstract

This paper presents the output of our fifth annual horizon-scanning exercise, which aims to identify topics that increasingly may affect conservation of biological diversity, but have yet to be widely considered. A team of professional horizon scanners, researchers, practitioners, and a journalist identified 15 topics which were identified via an iterative, Delphi-like process. The 15 topics include a carbon market induced financial crash, rapid geographic expansion of macroalgal cultivation, genetic control of invasive species, probiotic therapy for amphibians, and an emerging snake fungal disease.

## Highlights

• This is the fifth in our annual series of horizon scans published in *TREE*.
• We identify 15 issues that we considered insufficiently known by the conservation community.
• These cover a wide range of issues. Four relate to climate change, two to invasives and two to disease spread.
• This exercise has been influential in the past.

## Reasons for scanning the horizon

Horizon scanning is the systematic search for, and examination of, potentially significant medium- to long-term threats and opportunities that are not well recognized within a particular field [1]. The focus of this horizon scan is conservation, and it comprises the fifth in a series of annual assessments 2, 3, 4, 5. Early identification of plausible future issues for conservation could reduce the probability of sudden confrontation with major social or environmental changes, such as the introduction of biofuels in the USA, Canada, and the European Union (EU) 1, 6, 7. Horizon scanning may also raise awareness and provide momentum to scientific, technological, and policy innovation.

The use of horizon scanning in conservation is increasing. A parallel series of exercises has identified forthcoming changes in legislation that are likely to affect countries in the UK, the rest of the EU, and elsewhere 8, 9, 10. We are aware of planned horizon scans on environmental change in Antarctica, management of zoos and aquaria, and wetlands. High-priority questions for research and policy-making have also been identified for agriculture and natural resource management at national and international levels 11, 12, 13. Such assessments of research and policy questions can be stimulated by horizon scans.

The utility of horizon scans, individually or in aggregate, can be assessed in part by whether they succeeded in identifying topics that became major issues within a specified time frame, in our case years to decades. Several environmental topics identified by horizon scans published in *TREE* over the past 4 years, such as artificial life, synthetic meat [2], and hydraulic fracturing [3], have indeed moved from the horizon to the present, and are now widely discussed and better understood (see, for example, [14]). The probability of a horizon issue becoming current may sometimes be low, but the issue nevertheless warrants identification if its effects could be substantial. Thus, it is not expected that all topics identified in a horizon scan will become prominent. An alternative metric of the value of horizon scanning could be a reduction in the proportion of emerging issues that were not identified.

If forthcoming major issues are identified, then another measure of the importance of horizon scanning is the extent to which it encourages researchers to study emerging topics, and policy makers and practitioners to be vigilant and consider their responses should the issues be realized (e.g., [15]). A mechanism for increasing the utility of a horizon scan to serve this role is to use the scan to guide future strategies or funding. For example, the Intergovernmental Platform on Biodiversity and Ecosystem Services (IPBES), which held its first plenary meeting in 2011, seeks to lead the global scientific community in responding to major environmental changes. It has been suggested that IPBES use horizon scanning to develop its work program [16], and Germany is already doing so to guide its input to the Platform.

The outputs of horizon scans can directly inform policy-making. For example, during the continuing development and implementation of the Food Safety Modernization Act in the USA, federal regulators became aware that sterile farming could affect natural communities and ecosystems. The regulators were informed in part by identification of sterile farming in our 2012 horizon scan [4] and by subsequently published analyses of the potential environmental effects of sterile farming ([17]; personal communication Gennet 2013). Thus, arguably the greatest value of horizon scanning is stimulating action to prevent a plausible threat from being realized.

## Identification of issues

The methods used in this horizon scan, described in [18], were the same as in our previous scans. The inclusive, transparent, and structured communication process we adopted is a modification of the Delphi technique, which was developed for systematic forecasting 19, 20.

The 20 core participants in the horizon scan (the authors) include professional horizon scanners and experts in disciplines relevant to conservation science who collectively are affiliated with organizations with diverse research, management, and communications mandates. Each participant, independently or in consultation with others, suggested two or more issues that they considered to be emerging, of global scope or relevance, and not widely known within the conservation community. At least 369 individuals actively contributed to the generation of ideas, of whom approximately 150 were reached through broad solicitation of the expert commissions of the International Union for Conservation of Nature. Short (approximately 200-word) descriptions of the resulting 81 issues were distributed to all core participants, who scored each issue from 1 (well known, or poorly known but unlikely to have substantial environmental effects) to 1000 (poorly known and likely to have substantial environmental effects). Scores were converted to ranks and the rank for each issue averaged across participants. The 35 issues with highest mean ranks, an additional four issues inadvertently omitted from the first round of scoring, and four issues that participants thought warranted further consideration were retained for further discussion. For each of these 43 issues, two participants who had not suggested the issue further researched its technical details and assessed its probability of becoming prominent.

In September 2013, the core participants met in Cambridge, UK. Each issue was discussed in turn. The person who suggested the issue was not among the first three people to discuss it. Participants then independently and confidentially scored the issues again as described above. The 15 issues with the highest mean ranks are presented here. The order of presentation does not reflect mean rank, but related issues are placed together.

## The issues

### Response of financial markets to unburnable carbon

There is an incompatibility between current stock market valuation of the fossil fuel industry, which is based on known and projected fuel reserves, and governmental commitments to prevent a rise in global average temperature of more than 2°C above pre-industrial levels [21]. It has been suggested that the carbon budget from 2013 through 2050 should not exceed approximately 600–900 Gt CO_2_ for the probability of a >2°C temperature increase to remain ≤20% 21, 22, 23. By comparison, the carbon embedded in the known global coal, oil, and natural gas reserves amounts to 2860 Gt CO_2_. Reliable reserves held by companies listed on stock exchanges around the world already amount to 762 Gt CO_2_. Nonetheless, the industry invests approximately US$650 billion/yr in exploring new fossil-energy sources and in new extraction methods [21]. For the 200 largest listed companies in the world, these reserves have an estimated market value of US$4 trillion. However, this market value may decline sharply if fossil fuels are not burned because regulations are developed to comply with international agreements on emission limits. If investors and regulators do not address these trade-offs, governments may be forced to choose between preventing further climate change (risking a financial crisis) or preventing a financial crisis (risking further climate change).

### Extensive land loss in Southeast Asia from subsidence of peatlands

In recent decades, over 10 million hectares of coastal and lowland peat swamp forest in Southeast Asia have been converted to drainage-based agriculture and plantations [24], resulting in rapid peat oxidation 25, 26. Combined with compaction and shrinkage of peat, this conversion will lead to subsidence of almost all lowland peat in Indonesia and Malaysia by as much as 2.5 m within 50 years and 4 m within 100 years [26]. In coastal areas and floodplains, subsidence could increase the probability of inundation well beyond projected effects of climate change, such as rising sea levels. In the Netherlands, long-term drainage of peat for agriculture has led to subsidence of extensive areas to 6–8 m below sea level. Costly systems, such as dykes, and pump-operated drainage systems have been necessary to avoid inundation of densely populated, economically productive areas 27, 28. The high intensity of rainfall in Southeast Asia means that mitigation measures applied in the temperate zone may not be effective and that extensive areas will be flooded. Yet, the focus of research, policy, and planning has been subsidence and associated flooding in urban areas [29]; peatland subsidence in rural areas has not been taken into account.

### Carbon solar cells as an alternative source of renewable energy

Silicon-based solar photovoltaic cells are becoming a promising source of renewable energy as their installation costs decrease, potentially reducing the magnitude of climate change. However, construction requires rare conductive metals and indium tin oxide. Researchers have now built the first carbon solar cell, in which both the active layer and electrodes are carbon [30]. In a carbon solar cell, silver and indium tin oxide are replaced by graphene and single-walled carbon nanotubes, which are efficient conductors and light absorbers. Methods for producing carbon nanotubes, graphene, and fullerenes have also advanced substantially. Coating can be applied from solution, thus enabling the cells to be more flexible compared with rigid silicon cells. The thin-film cells that have been built with a carbon-based active layer are prototypes with relatively low efficiency (approximately 1% of solar energy is converted to electrical energy, compared with the 20% solar conversion efficiency of silicon-based solar cells; [31]). However, mass production could result in cheaper cells that could be installed on land (including on buildings) or in water, or worn by humans. The area necessary to operate carbon cells is smaller per unit energy than that of operating photovoltaic arrays [32]. Carbon solar cells have the potential to reduce demands for other means of generating electricity that generate greater quantities of greenhouse gases, to provide electricity in regions where other sources of electric power are not available, and to power remote-tracking or data-recording devices.

### Rapid geographic expansion of macroalgal cultivation for biofuels

Algae have long been harvested for human consumption and, more recently, have been used in a range of food, biotechnology, cosmetics, and other industries. Research into industrial-scale macroalgal use as biofuel began during the 1970s [33], and there is now evidence that initial challenges of cost and efficiency are being overcome 34, 35. Potential marine-based biofuel capacity could be up to six times that of terrestrial biofuels [36] and both governments and industry are now investing in trials and modest expansion in, for example, Australia, Denmark, Ireland, Norway, Portugal, and the UK (e.g., http://www.biomara.org and http://www.seaweedenergysolutions.com). Opportunities for expansion of macroalgal production in developing countries could be considerable [37]. Unlike many biofuels, macroalgae do not compete for agricultural space or for freshwater. However, macroalgal cultivation still requires considerable space in often intensively used coastal waters, and may suppress benthic communities, such as seagrasses. One future scenario developed by the UK Government included over 470 000 ha of macroalgal culture by 2050 [38]. Although macroalgal cultivation may create competition with other sectors, there could be synergies with both offshore production of renewable energy and fish cultivation [39]. The ecological effects of extensive production are as yet unknown, but could change the structure, composition, and function of marine environments through shading, smothering, and the alteration of nutrient regimes and trophic pathways.

### Redistribution of global temperature increases among ecosystems

The steady increase in the concentration of greenhouse gases has resulted in temperature increases of approximately 0.8°C at the surface of the Earth over the past 150 years. However, the rate of surface temperature increase has slowed over the past decade. The change in rate could be a result of typical climate variability, but there is some evidence that the heat has been redistributed and much of the warming has occurred in the ocean at depths below 700 m, rather than at the surface 40, 41, 42. Correction of the data for changes in measurement methods does not remove the temporal trend. The redistribution, which is thought to result from changes in surface winds over the past decade, raises uncertainties about the capacity of the deep ocean to absorb heat energy, potential feedbacks on the melting of polar ice sheet due to upwelling of warmer ocean water, and the effect of redistribution on ocean currents. Redistribution may also reduce the projected magnitude of responses of organisms that live on land or near the sea surface to increases in global temperature. Furthermore, the likely effects of increases in ocean temperature on deep sea and other marine species are unknown. Policy makers could erroneously interpret the reduced rate of surface temperature increase as a lower climate forcing from greenhouse gas emissions rather than a reflection of the complex interactions of natural variability and global resilience to greenhouse gas emissions. This misinterpretation may lead to calls to slow policy initiatives aimed at reducing greenhouse gas emissions.

### High-frequency monitoring of land-cover change

As new satellites and remote sensors are deployed, the quantity and quality of data available at low to no charge will increase dramatically. Simultaneously, free or inexpensive data from established aerial imagery and satellite systems will enable temporally consistent monitoring of land cover. The moderate resolution imaging spectroradiometer (MODIS) and Landsat acquire global images every 7–16 days and privately operated satellites, such as the Disaster Monitoring Constellation 3 (Surrey Satellite Technology Ltd), due to be launched in 2014, have the potential for daily acquisition of images over smaller areas. This means that it is now theoretically possible to monitor land cover in near real-time. However, data processing is still labor intensive and image quality affects interpretation. Some land-cover types, such as tropical rain forest, urban, and certain crops, are easy to monitor (e.g., 43, 44) whereas others, such as native grasslands and aquatic types, are more difficult. Frequent, fine-resolution monitoring is currently most feasible at relatively small spatial extents (e.g., tens of km) and for a subset of land-cover types, in particular those with trees. The ability to monitor land-use change at high temporal frequency over larger extents could inform management of international efforts to mitigate climate change, or certification of sustainable farmed food or fuel products in global supply chains. Anticipated advances, such as automated image processing, the differentiation of nonforest land-cover types, and the identification of features at finer resolutions, will make this technology increasingly useful.

### Reaccelerated loss of wild rhinoceroses and elephants

Organized crime syndicates are driving a dramatic acceleration in the loss of elephants and rhinoceroses across Africa and Asia, undermining decades of concerted conservation action. The number of forest elephants (*Loxodonta cyclotis*) in central Africa declined by 62% between 2002 and 2011, and the geographical range of the taxon decreased by 30% [45]. High human population density, intensive and/or illegal hunting, absence of law enforcement, poor governance, and proximity to expanding infrastructure are fuelling this decline [45]. Ivory poaching has increased dramatically over the past decade [46], largely due to the increasing price and demand for ivory in China 47, 48. Even in well-protected reserves, threats to the species have escalated [48]. The International Union for Conservation of Nature (IUCN) Species Survival Commission estimated that, at the start of 2013, there were 20 405 white rhinoceros (*Ceratotherium simum*) and 5055 black rhinoceros (*Diceros bicornis*) across Africa; by September, 613 rhinoceros had been poached for their horn in South Africa alone, many more than in 2011 or 2012. The retail price of rhinoceros horn in user countries still exceeds that of gold [49]. With growing wealth in Asia and as the species become scarcer, their value might increase even further and escalate speculation for illegally traded commodities, such as elephant ivory and rhinoceros horn.

### Increasing scale of eradications of non-native mammals on islands

There may soon be a step change in the potential spatial extent of eradications of non-native invasive mammals on islands as expertise and techniques improve. Such methods include new toxins (e.g., para-aminopropiophenone), and the use of global positioning systems to deliver baits aerially [50] (http://eradicationsdb.fos.auckland.ac.nz/). Recent or current extensive programs that have been successful include the eradication of rabbits (*Oryctolagus cuniculus*), black rats (*Rattus rattus*), and feral domestic cats (*Felis catus*) from Macquarie Island (12 800 ha), and goats (*Capra aegagrus hircus*) from Aldabra (15 400 ha) and Isabela (463 000 ha). The eradication of reindeer (*Rangifer tarandus*), brown rats (*Rattus norvegicus*), and house mice (*Mus musculus*) from South Georgia (360 000 ha) is underway. There are also recent examples of unsuccessful eradication campaigns, such as Pacific rats (*Rattus exulans*) from Henderson Island (4300 ha; http://eradicationsdb.fos.auckland.ac.nz/). The potential of new toxins and tools (e.g., lures, rechargeable traps, and bait dispensers) to attract and eradicate non-native mammals at low densities is being assessed. Given such advances, it has been proposed to eradicate all non-native invasive mammalian predators [rats, stoats (*Mustela erminea*), and possums (*Trichosurus vulpecula*)] from the entire New Zealand archipelago, starting with Rakiura-Stewart Island (>174 000 ha) [51]. A more controversial but highly publicized campaign (‘Cats to Go’; http://garethsworld.com/catstogo) encourages New Zealand citizens to protect native birds by ceasing to keep domestic cats as pets. Spatially extensive eradications increase the probability of conserving native species, but the risks of failure and public opposition may also grow as the extent of eradications increases and encompasses places with large human populations (e.g., [52]).

### Self-sustaining genetic systems for the control of non-native invasive species

Control methods for some invasive species are highly effective, but for some other species, current control methods are either ineffective or nonexistent. Genetic control techniques that transmit heritable elements to make individuals sterile are developing rapidly for disease vectors with substantial effects on human health, such as *Aedes* mosquitoes 3, 53. Some methods being proposed and developed for insects spread genes through a population despite the genes conferring a reproductive disadvantage. In principle, the use of such genetic systems would reduce the need for periodic extensive and expensive release of carriers of the desired traits. This makes the methods more applicable to eradication or control of large, well-established populations of non-native invasive species, which could increase the probability of conserving native species. An example of such methods is the proposed use of homing endonuclease genes [54], which replicate in the genome using the DNA repair mechanisms of the cell. These genes could be used to cause mortality or sterility in a particular life stage or sex or under a particular set of conditions. Another example is cytoplasmic male sterility in plants, where genes are transmitted through mtDNA [55]. Researchers are pursuing theories and models of self-sustaining genetic control of non-native invasive fishes [56] and plants [55]. Beyond effects on the target non-native species, the potential environmental effects of these systems, such as the unintended transport or dispersal of target species to other locales, horizontal gene transfer, and unforeseen ecological persistence of heritable control elements, have not been investigated in detail.

### Probiotic therapy for amphibians

Many amphibian populations in relatively pristine habitats are in decline or are becoming extirpated due to the skin disease chytridiomycosis [57]. Probiotic therapy through bioaugmentation is now emerging as a potential solution for mitigating this disease 57, 58, 59. The microbiome, the bacteria, fungi, and viruses that live within and upon every organism, has become a growing area of human health research, but relatively little attention has been paid to the nonhuman microbiome [60]. Bioaugmentation could both facilitate reintroduction of amphibians to areas from which they have been extirpated and reduce the magnitude of declines in areas not yet affected by chytridiomycosis [57]. Although the concept of probiotic therapy is promising, laboratory and field experiments on treatment of amphibians with probiotic baths have yielded mixed results, and the method has not yet been applied over large natural areas 57, 58, 59, 61. Potential environmental effects of bioaugmentation on nontarget amphibians and other taxonomic groups are not well known. Similarly, the effects of human activities on plant and animal microbiomes, and upon the emergence and transmission of disease [62], especially in relation to the release of antibiotics, are poorly studied.

### Emerging snake fungal disease

Snake fungal disease (SFD) is an emerging disease of wild snakes in the eastern and midwestern USA. The likely etiologic agent is the fungus *Ophidiomyces* (formerly *Chrysosporium*) *ophiodiicola*, which is consistently associated with the often-fatal skin, face, and eye lesions characteristic of the disease [63]. SFD was documented in captivity [64] but infrequently reported from the wild before 2006; however, its incidence appears to be increasing [63]. The disease has now been documented across nine US states and in seven species, and is likely to be more widespread. Although limited long-term monitoring and the cryptic, often solitary nature of snakes, make it difficult to characterize definitively mortality rates, transmission patterns and population-level effects, these may be substantial. It appears that, during 2006 and 2007, SFD contributed to a 50% decline in the abundance of timber rattlesnake (*Crotalus horridus*) in New Hampshire. However, the disease has been observed in regions without suspected snake declines [63]. Indirect infection via environmental exposure rather than proximate contact may be possible [65]. In light of other recently emerged fungal diseases that have caused precipitous and spatially extensive population declines [66], particularly chytridiomycosis in amphibians and white nose syndrome in bats, SSFD may warrant an increase in research and monitoring. Precautions, such as instrument decontamination, may reduce its spread.

### Polyisobutylene as a marine toxicant

Polyisobutylene (PIB) is a gas-impermeable synthetic rubber that is manufactured and used globally to produce lubricants, adhesives, sealants, fuel additives, cling-film, and chewing gum. Global consumption is projected to increase by approximately 40% to 1.2 million tons/yr by 2017 [67]. Under the Marine Pollution Regulation (MARPOL) Convention, PIB can be discharged in limited amounts under certain conditions*. PIB is hydrophobic: on contact with water, it becomes waxy and sticky, floating either on or near the surface. It has been found to adhere to the bodies of birds, especially diving species, causing immobilization, hypothermia, starvation, and eventually death. At least four releases of PIB have led to mass seabird deaths near European coasts 68, 69. Little is known about decomposition of PIB or its interactions with other additives or cleaning agents used in ship-tank washing. Questions about the effects of PIB discharge were raised at the Marine Environment Protection Committee 2013 session of the International Marine Organizations [70]. In response, the International Parcel Tankers Association suggested recent releases were not standard operating procedure and, thus, argued against regulation. However, international monitoring and reporting is limited, and the nature and extent of environmental effects remain unknown.

### Exploitation of Antarctica

Pressure for exploration and subsequent exploitation of the minerals and hydrocarbons of Antarctica is increasing. The Antarctic Treaty precludes ‘mineral resource activities’ until 2048 and states, ‘Antarctic mineral resource activities means prospecting, exploration or development, but does not include scientific research activities within the meaning of Article III of the Antarctic Treaty.’ Although prospecting is prohibited by the treaty, several countries have substantially increased their geological exploration, which has been interpreted widely as a step towards prospecting. In 2011, Russia stated its intention to conduct ‘complex investigations of the Antarctic mineral, hydrocarbon and other natural resources’ as part of its research on the Antarctic continent and surrounding seas to 2020 [71]. These actions conflict with at least the spirit of the Madrid Protocol, yet none of the other 48 signatories to this protocol expressed opposition [72]. China is building a new Antarctic base that, according to the western press, does not have the scientific justification required by the Antarctic Treaty [71]. It appears likely that exploitation will occur, and access to the continent is increasing in response to, for example, loss of glaciers on the Antarctic Peninsula. Given that Antarctica is so remote and ice shelves cover extensive seas, removal of oil spills or releases of toxicants and subsequent recovery is likely to take longer than elsewhere in the world and, thus, the environmental effects may be greater.

### Expansion of ecosystem red listing

In 2008, the IUCN Commission on Ecosystem Management launched the development of the scientific foundations for a Red List of Ecosystems [73] to complement the long-established IUCN Red List of Threatened Species (http://www.iucnredlist.org). Categories and criteria have been published [74] with the aim of applying them across all ecosystems by 2025 [73]. In parallel, interest in the implementation of risk assessments at national and regional levels is growing rapidly. Ecosystem red listing of the terrestrial ecosystems of the Americas is underway [75], as are national-level analyses in seven Mesoamerican and South American countries. Proposed expansion of ecosystem red lists to Europe, New Zealand, parts of Africa, Oceania, Asia, and other regions is gaining traction. The publication of these assessments could lead to major additional resource allocations by funding agencies because the assessments will provide greater clarity on which ecosystems are most threatened. However, concerns exist that policy-makers inadvertently may deemphasize ecosystems where original structure, composition, or function are documented to have changed dramatically. Additionally, because ecosystem red listing potentially can be carried out at multiple spatial scales [74], assessors may come under pressure to choose units of assessment on the basis of achieving political objectives rather than on the basis of ecological function.

### Resurrection of extinct species

Developments in synthetic biology raise the possibility that extinct species could be resurrected or reconstructed [76]. High-profile candidates include woolly mammoth (*Mammuthus primigenius*), passenger pigeon (*Ectopistes migratorius*), and thylacine (*Thylacinus cynocephalus*). Three potential methods are back-breeding, cloning, and genetic engineering; only cloning has the potential to produce an exact copy of an extinct species, although even in this case the embryo would develop in a foster species and, thus, the product might not be identical. Rapid sequencing could enable reconstruction of the genome of an extinct species from DNA fragments. Thus, it would be theoretically possible either to engineer genetically the chromosomes (and create embryonic cells by cloning) or, more likely, to modify the genome of an extant species that has a close phylogenetic relation to the extinct species. Resurrection could return extinct keystone species to ecosystems and, even if individuals were restricted to captive populations, provide tools for outreach and education. However, focus on a small number of iconic extinct species might divert attention and resources from preventing contemporary extinctions and maintaining and restoring ecosystems [77]. The viability, ethics, and safety of releasing resurrected species into the environment have not been fully explored [78].

## Discussion

As in previous annual horizon scans, the environmental effects of some of the issues that we identified this year may be valued by society (e.g., increasing scale of eradications of non-native mammals on islands or expansion of ecosystem red listing), whereas others may be considered undesirable (e.g., PIB or exploitation of Antarctica). Most are likely to have some positive and some negative effects, but inherent to horizon scanning is an inability to project trade-offs with high certainty.

Common horizon themes across recent years include emerging diseases, the ecological role of microbiota, nonrenewable and renewable energy, and under-recognized effects of human activities on the marine environment. For example, previous exercises identified the re-emergence of rinderpest in 2011; sterile farming in 2012, and links between autoimmune function and microbial diversity in 2013; mining in the deep ocean in 2012; and both seabed-located oil drilling and processing and rapid growth of concentrated solar power in 2013. We suspect that temporal thematic similarities reflect major drivers of changes in environmental status and the unsurprising tendency of many environmental scientists and decision makers to focus on these phenomena. In this horizon scan, we identified several climatic processes and responses that likely interact ecologically and socially, and may be synergistic. For instance, the global market for carbon may affect distribution of increases in temperature and incentives for further development of solar cells.

Any such exercise emerges from the knowledge and views of the participants [18] and so it is important to strive for these participants, and those colleagues consulted in turn, to reflect the geographical and disciplinary diversity of the conservation community. There also are different approaches to horizon scanning, such as use of software that systematically browses the internet to identify issues of increasing frequency [79] or identifying all the threats to an issue, such as threats to viability of migratory shorebirds [9].

A frequent topic of discussion during our in-person meeting was the trajectory of emerging issues. In general, issues move from new to emerging to widely known to acted upon. Horizon scanning is the search for issues that are new and emerging, not those that are widely known and certainly not those that already are being acted upon. Some issues, such as SFD, clearly are new. However, many of the issues we identified are complex and longstanding. New aspects can emerge from general issues that are widely known and acted upon. For example, we selected the reaccelerated loss of wild rhinoceroses and elephants because current substantial declines follow a period of effective conservation, reduced pressure, and increases in population size. Conversely, we discussed effects of neonicotinoid pesticides on taxonomic groups other than bees (e.g., aquatic insects and birds), but did not include the pesticides as a horizon issue because policy action has recently been taken to temporarily suspend some uses of some neonicotinoids in the EU. In this context, we considered even emerging aspects of the issue to be reasonably well known 80, 81.

We also debated how to consider issues that gradually are becoming more likely to affect environmental status or decision-making. For example, we discussed numerous technological advances in small-scale monitoring, including those that track the behavior of large numbers of individual organisms. Although we accept that advances in tracking technology will revolutionize understanding of the natural world, we did not perceive a step change in the effects of such advances and, therefore, decided they did not warrant incorporation as horizon-scanning issues.

Evidence and certainty also affected our deliberations. For example, the geographic extent and taxonomic diversity of both snake declines and fungal outbreaks are poorly documented, and their effects on population- or species-level viability uncertain. A comparison could be made with the identification of chytridiomycosis in amphibians. By the time *Batrachochytrium dendrobatidis* was identified, it was widespread, and many amphibian populations were threatened or had been extirpated. During the early 1990s, although many experts were convinced that amphibians were declining worldwide, evidence tended to be anecdotal. Without long-term population data, it was difficult to conclude that something other than natural population fluctuations was occurring [82]. To be useful, horizon scanning must raise issues for which environmental effects are uncertain, and sometimes the issues will fade or be benign. Where malignancy arises, how the issue interacts with social or economic responses can determine the extent of its effects. Our intent is to stimulate a multidisciplinary, and globally visible, response to these alerts.

Horizon scanning is not intended to divert attention from contemporary phenomena known with high certainty to result in substantial environmental effects. Instead, it complements continuing decision-making and can inform strategic planning, long-term investments in research and monitoring, and implementation of adaptive management. It identifies risks and opportunities, encouraging policy makers and practitioners to develop policies and strategies that can respond to environmental and social change, and fosters innovation that can help strategic objectives.

Horizon scanning both yields a product and is a process. Some participants in previous horizon scans who initially were unfamiliar with the method have perceived changes in the disciplinary scope and evidence base of material that they canvas, whether in the peer-reviewed scientific literature or popular media. Perhaps most importantly, horizon scanning can encourage researchers, policy makers, and practitioners to engage in joint fact-finding, a process through which parties with potentially diverse interests collaboratively identify, define, and answer scientific questions that hinder development of effective policies [83].

## Footnotes

* Since the first submission of this article in September 2013, the working group of the International Marine Organization on the Evaluation of Safety and Pollution Hazards of Chemicals has proposed that high-viscosity PIB be recategorized, requiring ships to wash their tanks at a specialized facility. If approved, the requirement would take effect on 1 July 2016 (http://www.imo.org/MediaCentre/PressBriefings/Pages/47-PIB.aspx).

## References

[1] Sutherland W.J., Woodroof H.J. (2009). The need for environmental horizon scanning. Trends Ecol. Evol..

[2] Sutherland W.J. (2010). A horizon scan of global conservation issues for 2010. Trends Ecol. Evol..

[3] Sutherland W.J. (2011). A horizon scan of global conservation issues for 2011. Trends Ecol. Evol..

[4] Sutherland W.J. (2012). A horizon scan of global conservation issues for 2012. Trends Ecol. Evol..

[5] Sutherland W.J. (2013). A horizon scan of global conservation issues for 2013. Trends Ecol. Evol..

[6] Sutherland W.J. (2008). Future novel threats and opportunities facing UK biodiversity identified by horizon scanning. J. Appl. Ecol..

[7] Sutherland W.J. (2010). The identification of priority opportunities for UK nature conservation policy. J. Appl. Ecol..

[8] Sutherland W.J. (2011). What are the forthcoming legislative issues of interest to ecologists and conservationists in 2011?. Br. Ecol. Soc. Bull..

[9] Sutherland W.J. (2012). What are the forthcoming legislative issues of interest to ecologists and conservationists in 2012?. Br. Ecol. Soc. Bull..

[10] Sutherland W.J. (2013). What are the forthcoming legislative issues of interest to ecologists and conservationists in 2013?. Br. Ecol. Soc. Bull..

[11] Sutherland W.J. (2009). An assessment of the 100 questions of greatest importance to the conservation of global biological diversity. Conserv. Biol..

[12] Pretty J. (2010). The top 100 questions of importance to the future of global agriculture. Int. J. Agric. Sustain..

[13] Dicks L.V. (2013). What do we need to know to enhance the environmental sustainability of agricultural production? A prioritisation of knowledge needs for the UK food system. Sustainability.

[14] Fisk J.M. (2013). The right to know? State politics of fracking disclosure. Rev. Policy Res..

[15] Sutherland W.J. (2012). Enhancing the value of horizon scanning through collaborative review. Oryx.

[16] Vohland K. (2011). How to ensure a credible and efficient IPBES?. Environ. Sci. Policy.

[17] Gennet S. (2013). Farm practices for food safety: an emerging threat to floodplain and riparian ecosystems. Front. Ecol. Environ..

[18] Sutherland W.J. (2011). Methods for collaboratively identifying research priorities and emerging issues in science and policy. Methods Ecol. Evol..

[19] Rowe G., Wright G. (1999). The Delphi technique as a forecasting tool: issues and analysis. Int. J. Forecasting.

[20] Sutherland W.J. (2006). Predicting the ecological consequences of environmental change: a review of the methods. J. Appl. Ecol..

[21] Carbon Tracker Initiative (2013).

[22] Meinshausen M. (2009). Greenhouse-gas emission targets for limiting global warming to 2 degrees C. Nature.

[23] Meinshausen M. (2011). The RCP greenhouse gas concentrations and their extensions from 1765 to 2300. Clim. Change.

[24] Page S. (2009). Restoration ecology of lowland tropical peatlands in southeast Asia: current knowledge and future research directions. Ecosystems.

[25] Hooijer A. (2012). Subsidence and carbon loss in drained tropical peatlands. Biogeosciences.

[26] Couwenberg J., Hooijer A. (2013). Towards robust subsidence-based soil carbon emission factors for peat soils in south-east Asia, with special reference to oil palm plantations. Mires Peat.

[27] Kabat P. (2009). Dutch coasts in transition. Nat. Geosci..

[28] Leifeld J. (2011). Peatland subsidence and carbon loss from drained temperate fens. Soil Use Manage..

[29] Nicholls R.J., Cazenave A. (2010). Sea-level rise and its impact on coastal zones. Science.

[30] Ramuz M.P. (2012). Evaluation of solution-processable carbon-based electrodes for all-carbon solar cells. ACS Nano.

[31] Shea M.J., Arnold M.S. (2013). 1% solar cells derived from ultrathin carbon nanotube photoabsorbing films. Appl. Phys. Lett..

[32] Burke D.J., Lipomi D.J. (2013). Green chemistry for organic solar cells. Energy Environ. Sci..

[33] McHugh D. (2003).

[34] Suganya T. (2013). Production of algal biodiesel from marine macroalgae *Enteromorpha compressa* by two step process: optimization and kinetic study. Bioresour. Technol..

[35] Vanegas C.H., Bartlett J. (2013). Green energy from marine algae: biogas production and composition from the anaerobic digestion of Irish seaweed species. Environ. Technol..

[36] Florentinus A. (2008).

[37] Ecofys (2009).

[38] Department of Energy and Climate Change (2010).

[39] Roberts T., Upham P. (2012). Prospects for the use of macro-algae for fuel in Ireland and the UK: an overview of marine management issues. Mar. Policy.

[40] Balmaseda M.A. (2013). Distinctive climate signals in reanalysis of global ocean heat content. Geophys. Res. Lett..

[41] Held I.M. (2013). Climate science: the cause of the pause. Nature.

[42] Rosenthal Y. (2013). Pacific ocean heat content during the past 10,000 years. Science.

[43] Pittman K. (2010). Estimating global cropland extent with multi-year MODIS data. Remote Sens..

[44] Townshend J.R. (2012). Global characterization and monitoring of forestcover using Landsat data: opportunities and challenges. Int. J. Digit. Earth.

[45] Maisels F. (2013). Devastating decline of forest elephants in Central Africa. PLoS ONE.

[46] CITES (2012).

[47] Vigne L., Martin E. (2011). Consumption of elephant and mammoth ivory increases in southern China. Pachyderm.

[48] Wittemyer G. (2011). Rising ivory prices threaten elephants. Nature.

[49] Milliken T., Shaw J. (2012).

[50] Veitch C.R. (2011).

[51] Bell P., Bramley A. (2013).

[52] Oppel S. (2011). Eradication of invasive mammals on islands inhabited by humans and domestic animals. Conserv. Biol..

[53] Alphey L. (2013). Genetic control of *Aedes* mosquitoes. Pathog. Glob. Health.

[54] Burt A. (2003). Site-specific selfish genes as tools for the control and genetic engineering of natural populations. Proc. R. Soc. B.

[55] Hodgins K.A. (2009). Genetic control of invasive plants species using selfish genetic elements. Evol. Appl..

[56] Thresher R. (2013). Genetic control of invasive fish: technological options and its role in integrated pest management. Biol. Invasions.

[57] Bletz M.C. (2013). Mitigating amphibian chytridiomycosis with bioaugmentation: characteristics of effective probiotics and strategies for their selection and use. Ecol. Lett..

[58] Harris R.N. (2009). Skin microbes on frogs prevent morbidity and mortality caused by a lethal skin fungus. ISME J..

[59] Becker M.H. (2011). Towards a better understanding of the use of probiotics for preventing chytridiomycosis in Panamanian golden frogs. Ecohealth.

[60] Redford K.H. (2012). Conservation and the microbiome. Conserv. Biol..

[61] Smith R.K., Sutherland W.J. (2014).

[62] Keesing F. (2010). Impacts of biodiversity on the emergence and transmission of infectious diseases. Nature.

[63] Sleeman J. (2013).

[64] Rajeev S. (2009). Isolation and characterization of a new fungal species, *Chrysosporium ophiodiicola*, from a mycotic granuloma of a black rat snake (*Elaphe obsoleta obsoleta*). J. Clin. Microbiol..

[65] Allender M.C. (2011). *Chrysosporium* sp infection in eastern massasauga rattlesnakes. Emerg. Infect. Dis..

[66] Fisher M.C. (2012). Emerging fungal threats to animal, plant and ecosystem health. Nature.

[67] Merchant Research and Consulting Ltd (2012).

[68] Camphuysen C.J. (2010). Mystery spill of Polyisobutylene (C4H8)n off the Dutch coast affecting seabirds in March 2010. Seabird.

[69] Royal Society for the Protection of Birds (RSPB) (2013) Wildlife charities call for ban on discharge of sticky seabird killer. *RSPB News* April 2013

[70] Marine Environment Protection Committee (2013).

[71] Foucart, S. (2011) Pawns in play on Antarctic ice-cap. *The Guardian Online* 10 November 2011

[72] Millar, E. (2011) The beginning of the end for Antarctica? *The Bubble* 6 November 2011

[73] Rodríguez, J.P. *et al.* (2012) IUCN Red List of Ecosystems. *S.A.P.I.EN.S.* 5, http://sapiens.revues.org/1286

[74] Keith D.A. (2013). Scientific foundations for an IUCN Red List of Ecosystems. PLoS ONE.

[75] Rodriguez J.P. (2012). From Alaska to Patagonia: the IUCN Red List of the Continental Ecosystems of the Americas. Oryx.

[76] Sherkow J.S., Greely H.T. (2013). What if extinction is not forever?. Science.

[77] Pimm, S. (2013) Opinion: the case against species revival. *National Geographic* 12 March

[78] Redford K.H. (2013). Synthetic biology and conservation of nature: wicked problems and wicked solutions. PLoS Biol..

[79] Proulx R. (2013). Googling trends in conservation biology. Conserv. Biol..

[80] Goulson D. (2013). Review: An overview of the environmental risks posed by neonicotinoid insecticides. J. Appl. Ecol..

[81] Van Dijk T.C. (2013). Macro-Invertebrate decline in surface water polluted with imidacloprid. PLoS ONE.

[82] Pechmann J.H.K. (1991). Declining amphibian populations: the problem of separating human impacts from natural fluctuations. Science.

[83] Karl H.A. (2007). A dialogue, not a diatribe: effective integration of science and policy through joint fact finding. Environ. Sci. Policy.

